# Supplementary material for: Functional Connectivity of the Brain Across Rodents and Humans
Source: Front Neurosci. 2022 Mar 8;16:816331. doi: 10.3389/fnins.2022.816331 (PMC8957796; doi:10.3389/fnins.2022.816331)
Supplement: Supplementary file 1 [file Data_Sheet_1.docx]

**Supplementary Materials**


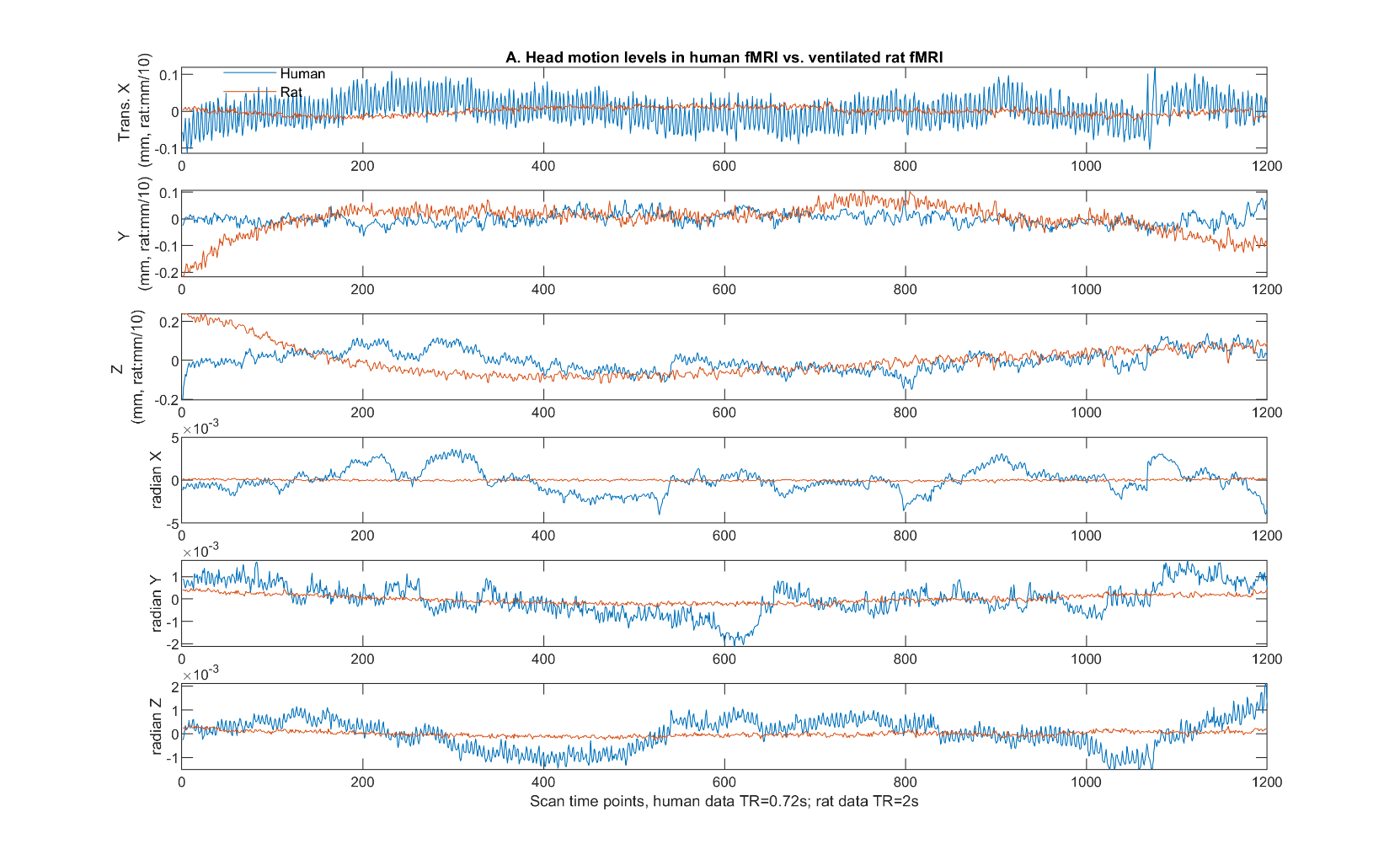


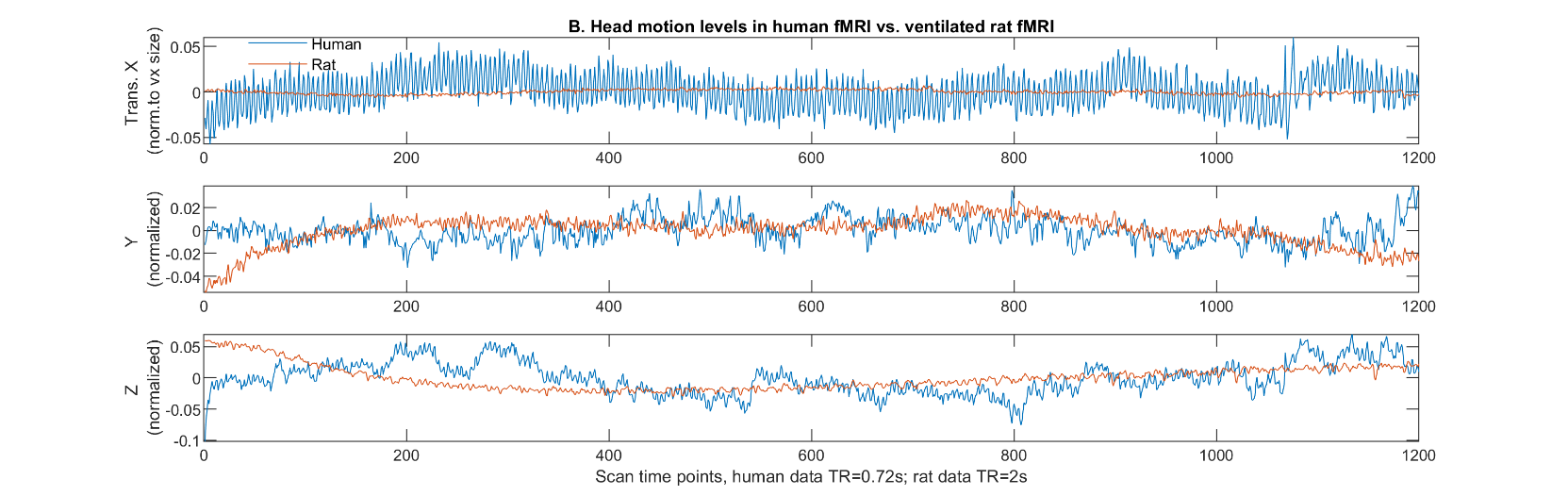


**Figure S1.** Head motion levels in a representative human rs-fMRI data set v.s. rat rs-fMRI data set. The blue lines show translation and rotation for a randomly selected subject from the 3T Human Connectome Project dataset (Van Essen et al. 2013); voxel size is 2mm isotropic. The red lines show translation and rotation for a randomly selected rat imaged under dexmedetomidine and low-dose isoflurane while paralyzed and mechanically ventilated; voxel size is 0.5mm isotropic (x10 scale in shown). The representative rat data are from (Pan et al. 2020). The full set of rat data will be deposited along with the project (<https://github.com/grandjeanlab/MultiRat>). The linear drifts were removed. Relative to human rs-fMRI data, the typical rodent rs-fMRI data has an extremely low level of head motion for both translation and rotation. Breath-related motions are common in human rs-fMRI but rarely seen in the data from ventilated rats. (A) shows the motion comparison in absolute translation distance and rotation (human, 2mm isotropic voxels; rat, 0.5mm isotropic voxels). The motion level in X direction translation (mean in mm +/- std), human: 0.0333+/-0.0227, Y, 0.0180+/-0.0140, Z, 0.0425+/-0.0004; rat: X 0.0009+/-0.0006, Y, 0.0038+/-0.0036, Z, 0.0062+/-0.0000; The motion level in X-direction rotation (mean in radian +/- std), human: 0.0011+/-0.0009, Y, 0.54e-3+/-0.41e-3, Z, 0.52e-3+/-0.36e-3; rat: X 0.0001+/-0.0001, Y, 0.16e-3+/-0.10e-3, Z, 0.09e-3+/-0.06e-3. Relative to the ventilated rat data, the human data may have significantly higher levels of rotation in all 3 directions, especially one direction with possibly breath waves. (B) shows the motion comparison in voxel relative sizes (normalized to absolute sized in each direction correspondingly). The motion level in X translation (mean+/-std), human: 0.0167+/-0.0114, Y, 0.0090+/-0.0070, Z, 0.0213+/-0.0155; rat: X, 0.0022+/-0.0014, Y, 0.0095+/-0.0089, Z, 0.0154+/-0.0116; The motion level in X direction (probably respiration-related) in human data may be almost one order higher than ventilated rat data. In the other two directions, the translation levels are similar or slightly better in the ventilated rat after linear drift removal (if higher-order drifts are removed, the rat head translations would be much smaller than the human translations).


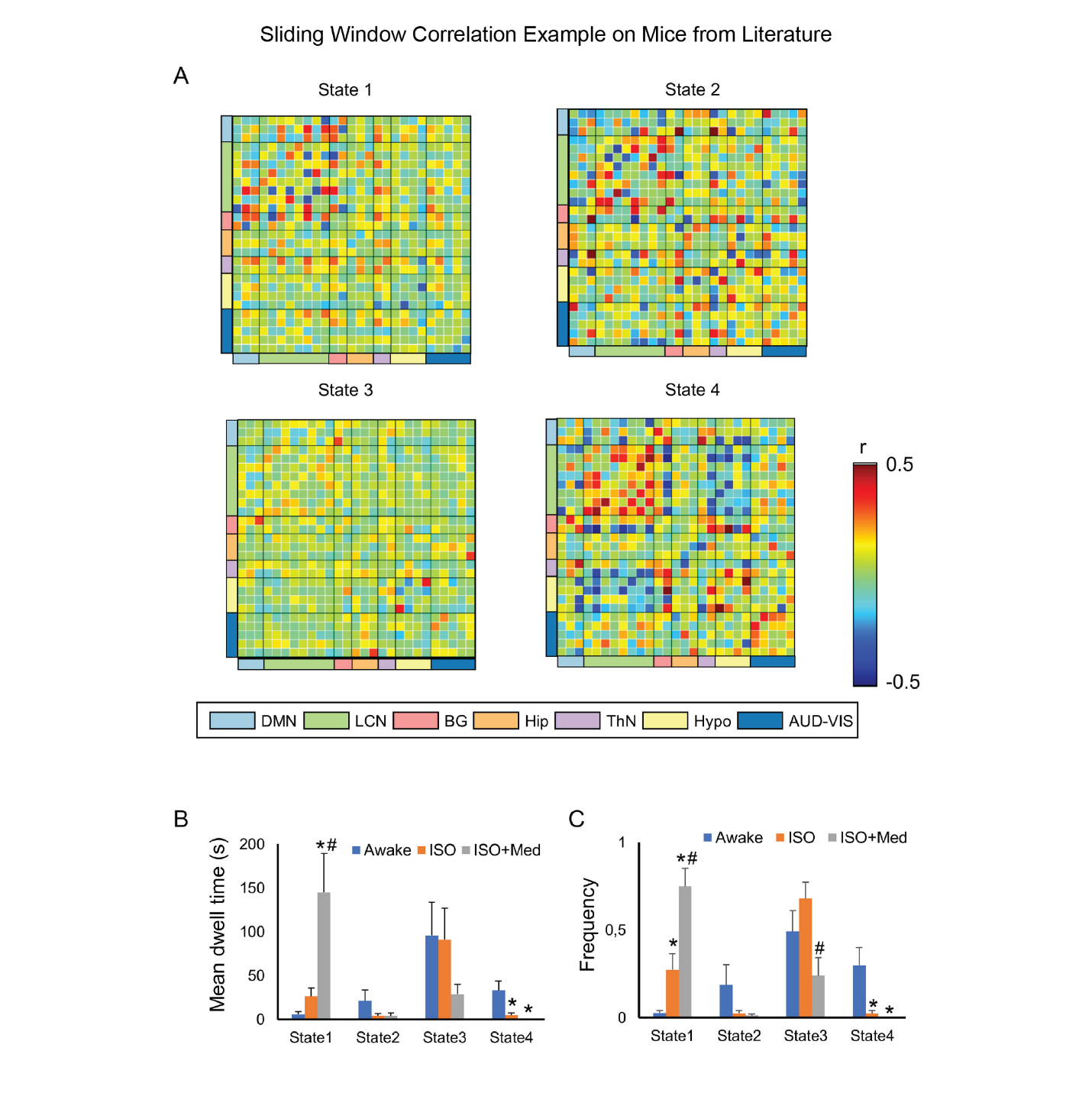


**Figure S2**: Example of windowed approaches for mice. Distinct brain states revealed by sliding window correlation are excerpted from (Tsurugizawa and Yoshimaru 2021) for mice. Four distinct mice brain states (A) were determined through the following procedures: First, using the fMRI ICA Toolbox (GIFT v3.0b http://trendscentre.org/software/gift/), fMRI timeseries for all three mice groups (i.e., 1 awake group (n=9), 1 isoflurane anesthesia group (n=9), and 1 medetomidine +isoflurane anesthesia group (n=9)) were extracted from ROIs that are determined by the ICA. These ICA determined ROIs cover the following brain networks: auditory-visual network (AUD-VIS); subcortical basal ganglion network (BG); default mode network (DMN); hippocampus (Hip);  hypothalamus (Hypo); lateral cortical network (LCN); thalamic network (ThN). Second, dynamic FC matrices were computed among these extracted fMRI timeseries using GIFT Dynamic FNC Toolbox (v1.0a). The window length was set to be 45 s (30 TRs), which slid in steps of 1.5 s (1 TR). Third, GIFT k-mean clustering was performed on these dynamic FC matrices to determine the optimal brain states. In this study, four optimal distinct brain states were found (K=4). (B and C) Averaged dwell time (B) and frequency of occurrence (C) of each brain state are also measured and compared between different groups using GIFT Dynamic FNC Toolbox (v1.0a). The dwell time measures the frequency of a state unchanged between the current and the next window, whereas the frequency of occurrence measures the number of windows in each state. Here, *p < 0.05 compared with the awake condition in each state, Tukey-Kramer test (df = 17). #p < 0.05 compared with the Iso condition in each state, Tukey-Kramer test (df = 17).


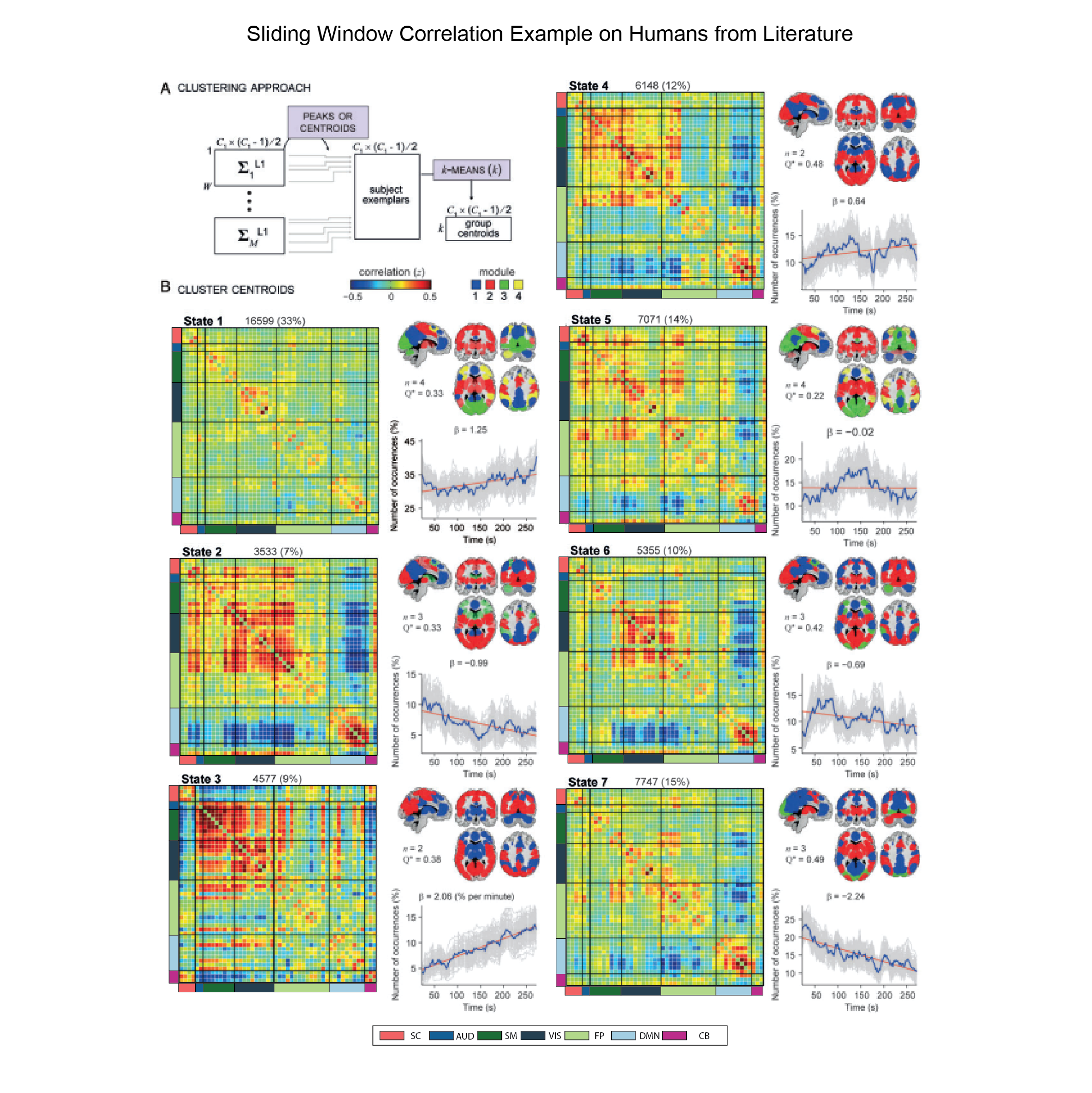


**Figure S3.** Example of windowed approaches for humans. Distinct brain states revealed by sliding window correlation are from (Fig. 5, Allen et al. 2012) for human brains. Seven distinct human brain states (B) were determined from the calculated sliding-window correlation matrices through the K-mean clustering procedure (A). More specifically, a group-level spatial ICA was first performed to determine the ROIs for all group data (405 healthy participants, 200 females, with age range 12–35 yro, and mean age at 21.0 yro). Second, dynamic FC matrices were computed among these extracted fMRI timeseries. The window length was set to be 44 s (22 TRs), which slid in steps of 2 s (1 TR). Third, GIFT K-mean clustering with 500 repetitions was performed on these dynamic FC matrices (as shown in (A)) to determine the optimal clusters and the cluster number. Each brain state is the K-mean cluster that was summarized by the cluster centroid (see the FC matrix as shown in (B)). In this human study, seven optimal distinct brain states were found (K=7). The number of occurrences of each state is shown as a function of time at the bottom right of each FC matrix. Finally, the Louvain algorithm was repeated on 100 bootstrap resamples within each cluster to obtain the modular structure for each brain state (top right). The module colors (red, blue, green, and yellow in the brain plots) were matched across states such that similar partitions share the same color. In order to compare with the mice brain networks in Figure S2, we added a similar set of color codes indexing networks that are homologous to the mice counterparts, which include DMN (light blue), frontoparietal (FP, light green), somatomotor (SM, dark green), auditory (AUD, blue), visual (VIS, dark blue), subcortical (SC, light red), and cerebellar (CB, violet). Note that the central executive network, which is known as FP in humans, is referred to as the LCN network in mice (Gozzi and Schwarz 2015).


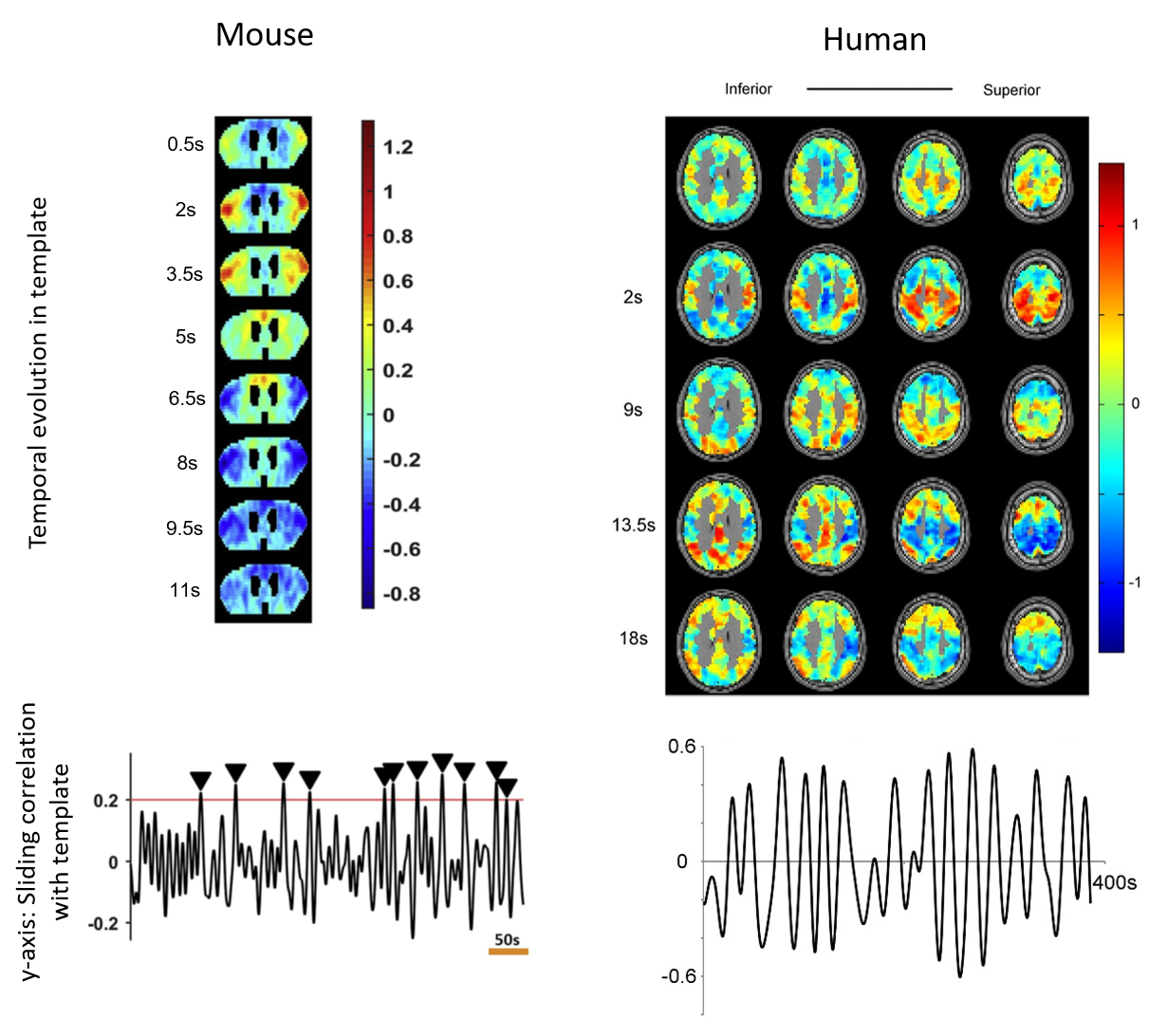


**Figure S4.** Example comparison of quasi-periodic patterns in mice and humans. The quasi-periodic patterns (QPPs; upper) and their correlation with the whole brain images across time (bottom) are from (Belloy et al. 2018) for mice (left) and from (Majeed et al. 2011) for humans (right). Specifically, the upper row illustrates the temporal evolution of the detected QPPs at an interval of 1.5s for mice (left) and an averaged interval of 5.67s for humans (right). For both mice and humans, alterations between DMN and task-positive network (TPN) are seen. For example, at the beginning stage of QPPs (t=2s), the TPN for both mice (the lateral cortical network) and humans (the attention network) shows a high signal when the signal from DMN is reduced. At an intermediate stage (t=5s for mice and t=9s for humans), a reduced TPN and an increased DMN are shown for both mice and humans. At the final stage (t=6.5s for mice and t=13.5s for humans) positive intensity in DMN and negative intensity in the TPN are observed. The signal intensity returns to baseline after some time, as seen in the last frame (t=11s for mouse and t=18 s for human). Separately, the bottom row illustrates the correlation value between the detected QPP and the whole brain images over time, namely ‘sliding correlation with template’ in the figure. The peaks, which are distributed in time, indicate that the QPP occurs repeatedly over time. For QPPs detection, both studies (Belloy et al. 2018; Majeed et al. 2011) used the pattern-finding algorithm developed in (Majeed, et al. 2011). Specifically, a window length of 12s (24TR, 1TR=0.5s) was preselected for a mice dataset of 11 male C57BL/6 J mice (22~24 weeks old) (Belloy et al. 2018), which revealed a non-redundant full-size QPP. In comparison, a window length of 20.1s (67TR with 1TR=0.3s) could achieve a non-redundant full-size QPP for a human brain dataset of 6 healthy adults (19~22 yro, 3 females) (Majeed et al. 2011).


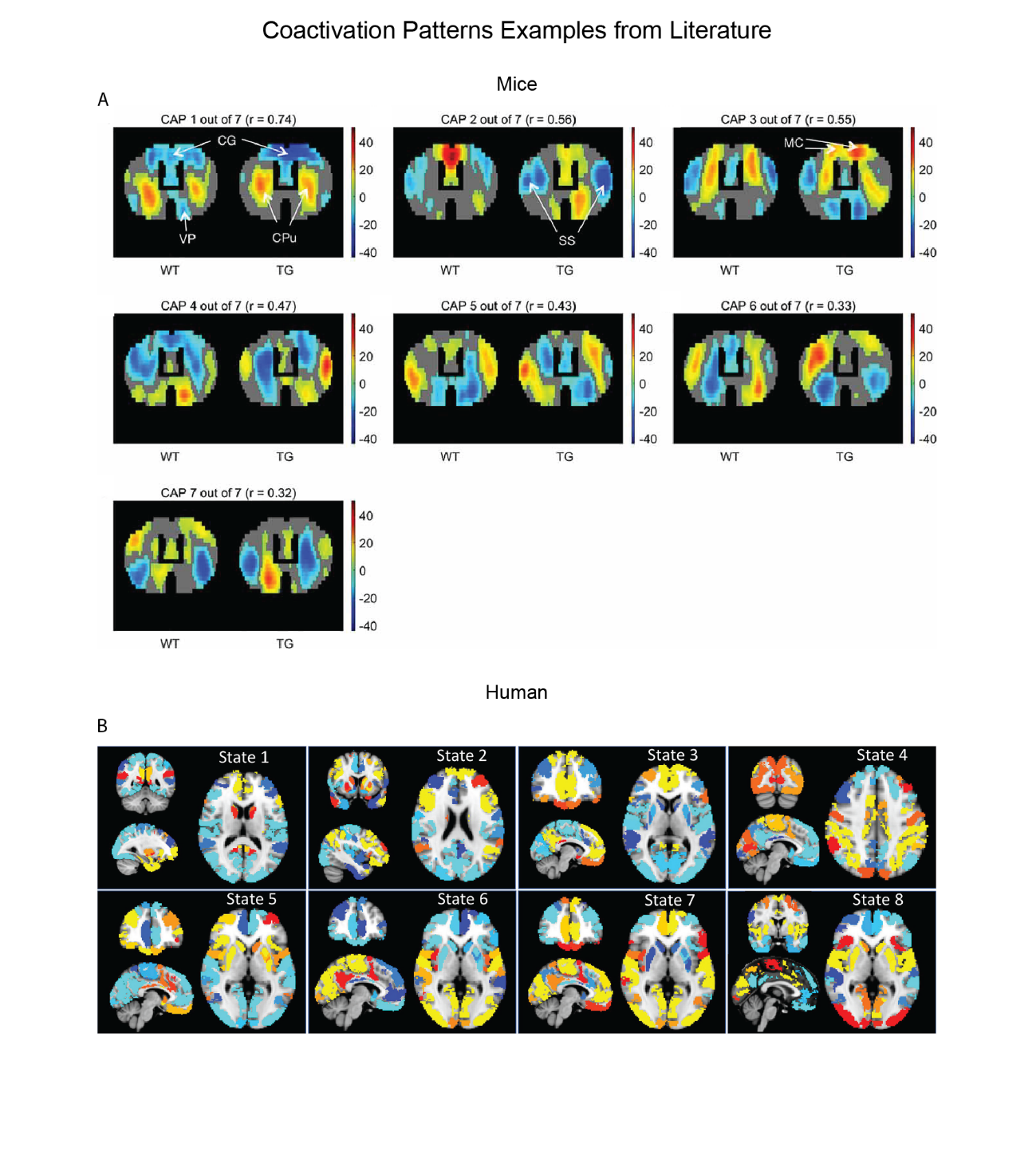


**Figure S5.** Example comparison of coactivations patterns (CAPs) in mice and humans. Brain states determined by CAPs are excerpted from (Adhikari et al. 2021) for mice (A) and from (Janes et al. 2020) for human brains (B). For the mouse study (A), 7 CAP brain states were contrasted between the Alzheimer’s disease model of 10 females at 18 months old (referring as the TG group) and 8 age-matched wild-type littermates (referring as the WT group). In particular, significantly (p < 1E-5; Bonferroni corrected) co-activated (T > 0) and co-deactivated (T < 0) brain voxels were tested by one-sample T-test as shown in (A). Distinctions in the following areas were detected between two groups: Cingulate cortex (CG), Caudate Putamen (CPu), Ventral Pallidum (VP), Somatosensory cortex (SS), Motor cortex (MC). For the human study (B), 8 CAP brain states were detected from resting brains of 462 Human Connectome Project subjects (281 females, 22~36 yro) (Van Essen et al. 2013). In particular, to identify the brain states, a K-means clustering analysis of CAPs was performed after an initial PCA dimensionality reduction step. The preselected number of clusters was explored from 4 up to 18, and the clustering solution was evaluated by silhouette scores. The optimal number of cluster K=8 was determined by the highest mean silhouette score. Warm colors represent activation (relative to within-state global average) while cool colors represent deactivation (relative to within-state global average) within each state.

**References**

Adhikari, Mohit H., Michaël E. Belloy, Annemie Van der Linden, Georgios A. Keliris, and Marleen Verhoye. 2021. “Resting-State Co-Activation Patterns as Promising Candidates for Prediction of Alzheimer’s Disease in Aged Mice.” *Frontiers in Neural Circuits* 14:91.

Allen, Elena A., Eswar Damaraju, Sergey M. Plis, Erik B. Erhardt, Tom Eichele, and Vince D. Calhoun. 2012. “Tracking Whole-Brain Connectivity Dynamics in the Resting State.” *Cereb Cortex* 24(3):663–76.

Belloy, Michaël E., Maarten Naeyaert, Anzar Abbas, Disha Shah, Verdi Vanreusel, Johan van Audekerke, Shella D. Keilholz, Georgios A. Keliris, Annemie Van der Linden, and Marleen Verhoye. 2018. “Dynamic Resting State FMRI Analysis in Mice Reveals a Set of Quasi-Periodic Patterns and Illustrates Their Relationship with the Global Signal.” *NeuroImage* 180:463–84.

Van Essen, David C., Stephen M. Smith, Deanna M. Barch, Timothy E. J. Behrens, Essa Yacoub, and Kamil Ugurbil. 2013. “The WU-Minn Human Connectome Project: An Overview.” *NeuroImage* 80:62–79.

Gozzi, Alessandro, and Adam J. Schwarz. 2015. “Large-Scale Functional Connectivity Networks in the Rodent Brain.” NeuroImage 127:496–509.

Janes, Amy C., Alyssa L. Peechatka, Blaise B. Frederick, and Roselinde H. Kaiser. 2020. “Dynamic Functioning of Transient Resting-State Coactivation Networks in the Human Connectome Project.” *Human Brain Mapping* 41(2):373–87.

Majeed, Waqas, Matthew Magnuson, Wendy Hasenkamp, Hillary Schwarb, Eric H. Schumacher, Lawrence Barsalou, and Shella D. Keilholz. 2011. “Spatiotemporal Dynamics of Low Frequency BOLD Fluctuations in Rats and Humans.” *NeuroImage* 54(2):1140–50.

Pan, W.-J., Sharghi, V. K., Zhang, X., & Keilholz, S. D. (2020). *(ISMRM 2020) Brain mechanism of anesthesia and sedation: fMRI functional connectivity study with minimized impact of physiological background noise in rats*. ISMRM. https://archive.ismrm.org/2020/3958.html

Tsurugizawa, Tomokazu, and Daisuke Yoshimaru. 2021. “Impact of Anesthesia on Static and Dynamic Functional Connectivity in Mice.” *NeuroImage* 241:118413.
